# Utilization of health management information systems for decision making among healthcare professionals in Ketu North Municipality, Ghana: a descriptive cross-sectional study

**DOI:** 10.11604/pamj.2025.51.66.41385

**Published:** 2025-07-04

**Authors:** Victor Zeng, Christopher Kankpetinge, Anthony Zunuo Dongdem, Philip Teg-Nefaah Tabong

**Affiliations:** 1 Health Information Unit, Volta Regional Health Directorate, Ghana Health Service, Ho, Ghana,; 2 Ghana Health Service, Municipal Health Directorate, Ketu North Municipal, Dzodze, Volta Region, Ghana,; 3 University of Health and Allied Sciences, Fred N. Binka School of Public Health, Ho, Ghana,; 4 University of Ghana, School of Public Health, Accra, Ghana

**Keywords:** Health worker, health management information system, utilization, decision-making, Ghana

## Abstract

**Introduction:**

Health Management Information System (HMIS) helps provide evidence-based decision-making, policy planning and adequate use of resources in the health system. The health system collects data but using it for decision making at the lower level remains a challenge. This study assessed the utilization of HMIS by sub-district health staff in Ketu North Municipality.

**Methods:**

a descriptive cross-sectional study was conducted among 219 health workers in six sub-municipalities using a structured questionnaire. Data was analyzed using SPSS Version 25 to determine association using Pearson's Chi-square/Fisher´s exact test. Multivariable logistic regression was used to identify factors associated with HMIS utilization.

**Results:**

healthcare workers who were given feedback on the data collected were 3.91 times likely to utilize HMIS for decision making compared to those who did not receive feedback (aOR: 3.91, 95% CI: 1.63 - 9.38, p=0.002). Healthcare workers who had low knowledge on HMIS were 2.56 times more likely to utilize HMIS for decision making compared to those healthcare workers with high knowledge on HMIS (aOR: 2.56, 95% CI: 1.04-6.31, p=0.041).

**Conclusion:**

the study revealed that healthcare professionals have limited knowledge on the use of HMIS. HMIS utilization was not affected by health facility type, employee category, or access to HMIS. Also, the study observed that health professionals who had access to feedback were more likely to utilize the information for decision-making purposes. Continuous training is recommended for healthcare staff to improve HMIS utilization.

## Introduction

Health information systems play a crucial role in the foundation of decision-making across the health system, influencing policy development, human resource development, health education and training, governance and regulation, health research, service planning, delivery, and financing [1,2]. Information technology is rapidly changing, and concepts and methods for making the best use of existing data for managing health services and resources are rapidly evolving in the world including sub-Saharan Africa [3-5]. Information at all management levels of health services, from the periphery to the central, is essential for patient/client management, health unit management, and planning and management in the health system [6-10]. The Ghana Health Service (GHS) was established in 1996 through Act 525 and is the largest autonomous national executive body responsible for the implementation of national policies related to health in Ghana [11]. It collaborates with the Ministry of Health (MOH) and operates a decentralized system at five levels: National, Regional, District, Sub-district, and community [12]. The GHS is mandated to collect, collate, and report routine services relating to health from all facilities across the country. Data from these facilities is processed into meaningful information useful to managers of health at all levels for planning, budgeting, and decision-making to improve health services management and delivery.

However, the process of obtaining routine service data from all facilities across Ghana has been the single most immediate challenge of the health sector, as it hinders health managers' ability to access timely data required for effective planning, budgeting, and decision-making [12,13]. In 2009, DHIMS2 was created, a web-based Health Management Information System (HMIS) designed to support district health administrations and health facilities at all levels in reporting and analyzing crucial health-related data [14,15]. To ensure the success of the integration of DHIMS2, staff in the GHS underwent training to acquire the necessary skills and capacity for utilizing the system and making informed decisions based on evidence rather than estimations [11,16-18]. Literature searches revealed that there has not been any study on the utilization of HMIS for decision-making in the Municipality. The findings may help health managers to assess the impact of HMIS utilization interventions implemented over the past years and help design strategies towards improving the utilization of HMIS for healthcare decision-making. This study aimed to assess the utilization of HMIS by sub-district health staff in Ketu North Municipality.

## Methods

**Study design and setting:** this study used a cross-sectional study design as described in earlier studies [19,20]. The study was conducted between November, 2019 and November, 2020 in Ketu North Municipal. Ketu North Municipal is one of the 18 Administrative Districts/Municipals in the Volta Region. It has its administrative capital at Dzodze and has six sub-districts with over five hundred health workers. The Municipal had a total projected population of 122,785 with 272 communities in the year 2020 [21].

**Study population:** the study participants were professional health workers trained from medical and allied health schools/colleges who had worked for at least six months. These individuals were the health care providers who maintained regular contact with clients and collected healthcare data [22]. Health workers who were on leave and those who had travelled were not included in the study. The sample size was calculated using a Cochran formula considering the following assumptions: a 95% level of confidence, a 5% margin of error and a design effect of 2. In addition, we utilized the p=78.5% from a similar study carried out in Kenya [20], which had a comparable demographic and study environment to the current study. A non-response rate of 10% was considered and a minimum sample size of 219 was eventually attained. Simple random sampling as used by Mboro [20], was used to select the study participants.

**Data collection:** the study employed a pre-tested self-administered questionnaire, which comprised five sections. The first two sections focused on health facility information, encompassing details like the facility's name, sub-district, ownership, and the interviewee's background information, including their responsibilities, category, and work experience. Sections three and four assessed the level of knowledge on Health Management Information Systems (HMIS), covering variables such as the definition of HMIS, the forms used in reporting, the ability to define and identify HMIS, the channels of reporting, and other related aspects. Additionally, these sections covered the utilization of HMIS, measuring variables such as the availability of HMIS in the sub-district, data entry into DHIMS-2 (District Health Information Management System) by either the facility or sub-district, and training on HMIS usage. The final section (part five) of the questionnaire explored variables influencing HMIS utilization, including the availability of a person responsible for data management at the sub-district level, data analysis practices, training on computer use, and the receipt of feedback. To collect the data, all study participants completed the questionnaires at their respective workplaces and subsequently returned them to the Principal Investigator.

### Definitions

***Health Management Information System (HMIS):*** a system design to collect, store, manage, and analyze health-related data to aid in healthcare decision-making, patient care, resource allocation, disease surveillance, and overall healthcare performance.

***Nurse:*** this includes Registered General Nurses, Enrolled Nurses, and Community Health Nurses.

**Data analysis:** data obtained was cleaned in Excel and analyzed using Statistical Package for Social Sciences (SPSS) Version 25. The categorical variables were summarized in frequencies and proportions. Pearson Chi square or Fisher´s exact test was used to determine the associations between the independent variables and HMIS utilization. Those independent variables (level of knowledge on HMIS, use of HMIS, and factors influencing HMIS) were categorized as low (< 50%), moderate (between 50% and 80%) and high (≥ 80%) and HMIS utilization for decision-making. The reliability index of this scale was calculated and it ranged between 0.67-0.86, indicating it was a good discrimination between study participants [23]. Multivariable logistic regression was used to identify factors associated with utilization of HMIS for decision making. This was done at two levels; crude and adjusted. All independent variables were added to the regression analysis model. However, only variables which were statistically significant (p<0.05) at the crude level were included in the model for the adjusted analysis.

**Ethical consideration:** approval to carry out this study was sought from the Graduate School of Catholic University College of Ghana and the Ethical Review Committee of Ghana Health Service (GHC-ERC054/10/19). Permission was obtained from the Municipal Director of Health Services Ketu North. Informed consent was sought from each participant before the interviews. Participation in the study was voluntary; hence, participants had the liberty to opt out at any stage if they did not wish to continue.

## Results

### General characteristics of the study population

The study involved 219 health care workers, with hospitals in the municipality having the highest rate (57.1%), Community-based Health Planning and Services compounds had the least (8.2%). Majority of the respondents, 57.1% were from Christian Health Association of Ghana (CHAG) facilities, while 29.7% were from government facilities and 12.2% from private facilities. The staff category consisted of 124 nurses (56.6%) forming the highest, followed by midwives, 32 (14.6%), and the least was Disease Control Officers, 5 (2.3%) (Table 1). Majority of the respondents had worked for five years or more (Table 2). The majority, 93.2% were knowledgeable about Health Management Information System (HMIS) with 88.2% defining it correctly, 47.9% had knowledge on some reporting forms, while 52.1% lacked knowledge. A good proportion of respondents, 170 (77.6%) knew about Hospital Administration and Management System (HAMS) and DHIMS-2 148 (67.6%) among HMIS types (Table 3).

**Table 1:** health facility ownership, type, staff category, access to HMIS and utilization in Ketu North Municipal, 2020

Independent variable	Utilisation of HMIS			Total n= 219	P-value
	Low n = 47 (%)	Moderate n = 69 (%)	High n = 103 (%)		
**Facility ownership**					0.175
Government	11 (23.4)	23 (33.3)	35 (34.0)	69 (31.5)	
CHAG (religious)	25 (53.2)	40 (58.0)	56 (54.4)	121 (55.3)	
Private	11 (23.4)	6 (8.7)	12 (11.7)	29 (13.2)	
**Facility type**					**0.044**
Hospital	30 (63.8)	29 (42.0)	62 (60.2)	121 (55.3)	
Health centre	6 (12.8)	10 (14.5)	20 (19.4)	36 (16.4)	
CHPS	3 (6.4)	8 (11.6)	7 (6.8)	18 (8.2)	
Clinic	8 (17.0)	22 (31.9)	14 (13.6)	44 (20.1)	
**Category of Health staff**					**0.001**
Medical Doctor	0 (0.0)	0 (0.0)	6 (5.8)	6 (2.7)	
Physician Assistant	1 (2.1)	7 (10.2)	0 (0.0)	8 (3.7)	
Midwife	5 (10.6)	11 (15.9)	16 (15.5)	32 (14.6)	
Nurse	31 (66.0)	38 (55.1)	55 (53.4)	124 (56.6)	
Health Information Officer	8 (17.0)	7 (10.2)	7 (6.8)	22 (10.1)	
Disease Control Officer	0 (0.0)	0 (0.0)	5 (4.9)	5 (2.3)	
Mental Health Officer	0 (0.0)	1 (1.4)	8 (7.8)	9 (4.1)	
Others	2 (4.3)	5 (7.2)	6 (5.8)	13 (5.9)	
**Access to HMIS in facility or sub-district**					**0.001**
Have access	19 (40.4)	35 (50.7)	94 (91.3)	148 (67.6)	
Have no access	28 (59.6)	34 (49.3)	9 (8.7)	71 (32.4)	

**Table 2:** association between Knowledge of respondents on utilization of HMIS in Ketu North Municipal, 2020 (n=219)

Independent variable	Utilisation of HMIS			Total (%)	P-value
	Low n= 47(%)	Moderate n= 69(%)	High n= 103(%)		
**Work experience of staff**					0.823
5 years and above	16 (34.1)	26 (37.7)	42 (40.8)	84 (38.4)	
3 years but <5 years	9 (19.1)	11 (15.9)	22 (21.4)	42 (19.2)	
1 year but <3 years	9 (19.1)	10 (14.5)	16 (15.5)	35 (15.9)	
6 months but <1year	13 (27.7)	22 (31.9)	23 (22.3)	58 (26.5)	
**Knowledge on reporting forms**					**0.001**
Able to mention at least a form	11 (23.4)	34 (49.3)	60 (58.3)	105 (47.9)	
Unable to mention a form	36 (76.6)	35 (50.7)	43 (41.7)	114 (52.1)	
**Knowledge on importance of HMIS**					**0.004**
Able to mention at least one importance	16 (34.0)	39 (56.5)	65 (63.1)	120 (54.8)	
Unable to mention any importance	31 (66.0)	30 (43.5)	38 (36.9)	99 (45.2)	
**Knowledge on reporting deadline**					**0.005**
2^nd^ of the new month	8 (17.0)	2 (2.9)	7 (6.8)	17 (7.8)	
3^rd^ of the new month	0 (0.0)	0 (0.0)	6 (5.8)	6 (2.7)	
4^th^ of the new month	3 (6.4)	5 (7.2)	14 (13.6)	22 (10.1)	
5^th^ of the new month	35 (74.5)	58 (84.1)	66 (64.1)	159 (72.6)	
None of the above	1 (2.1)	4 (5.8)	10 (9.7)	15 (6.8)	

P-value is significant if p < 0.05

**Table 3:** knowledge level of respondents on HMIS in Ketu North Municipal, 2020 (n=219)

Variable	Frequency	Percentage (%)
**Information or awareness of HMIS**		
Heard/know about HMIS	204	93.2
Not heard/know about HMIS	15	6.8
**Definition of HMIS**		
Correctly defined	180	88.2
Wrongly defined	16	7.8
No idea	8	3.9
**Knowledge on forms used to report HMIS**		
Know of forms to use	105	47.9
Don't known of forms to use	114	52.1
Ability to identify different types of HMIS*		
HAMS	170	77.6
DiHPART	82	37.4
Photo maker	1	0.5
DHIMS-2	148	67.6
**Practice of data entry at the sub-district level**		
Yes	171	78.1
No	48	21.9
**Availability of HMIS at the sub-district level**		
Yes	148	68.8
No	67	31.2
How health decisions are taken*		
Intuition	57	26.0
Evidence based	136	62.1
Directive from superiors	156	71.2
Cost	72	32.9
How data are used*		
Planning	179	81.7
Research	149	68.0
Deciding medical and drug supply and management	157	71.2
Day to day program management	162	74.0
Human resource management	150	68.5

Multiple responses*

### Factors associated with HMIS utilization for decision making

On respondents' level of knowledge and utilization of HMIS, it was observed that 103 (47.0%) of participants had high utilization, 69 (31.5%) showed moderate utilization, and 47 (21.5%) had low utilization. It was also revealed that, those with 5 or more years of work experience exhibited higher utilization rates (40.8% high, 37.7% moderate, and 34.1% low) compared to respondents with 1 to less than 3 years of work experience, who had the lowest utilization rates. With regard to knowledge on reporting forms, a greater proportion (52.1%) of the respondents could not mention any reporting form. A good proportion 159 (72.6%) of the respondents, however, knew the deadline of reporting to the next level. Respondents' knowledge on reporting forms (p= 0.001), the importance of HMIS (p= 0.004), reporting deadline (p = 0.005), and utilization of HMIS were found to have an association (Table 2).

On the ownership of facility, type of facility, category of staff, access to HMIS at facility, and HMIS utilization, it was observed that the rates of utilization of HMIS were higher among participants in CHAG (religious) facilities (high, 54.4%, moderate 58.0%, low, 53.2%) as compared to government and privately-owned facilities. By facility type, hospital staff formed the majority with high (60.2%), moderate (42.0%) and low (63.8%) HMIS utilization rates, whiles nurses were the most among the category of staff with high (53.4%), moderate (55.1%) and low (66.0%) utilization rates of HMIS. Also, 94 (91.3%) utilization rate was observed among respondents with access to HMIS at their facilities. There was a statistically significant relationship between health facility type (p= 0.044), category of staff (p= 0.001), access to HMIS in facility (p = 0.001) and utilization of HMIS. There was however no association between facility ownership and utilization of HMIS (p = 0.175).

Regarding healthcare decision-making using HMIS, the study revealed that a majority of the respondents 170 (77.6%) incorporated HMIS in planning their programmes of work and daily activities. Among the types of decisions made by the respondents concerning the use of HMIS, research-related decisions were the least common, with only 69 (31.5%) (Table 3). Multivariable logistic regression showed that factors associated with HMIS utilization for decision making included feedback on the data collected (aOR: 3.91, 95% CI: 1.63-9.38, p= 0.002), and low knowledge on HMIS (aOR: 2.56, 95% CI: 1.04-6.31, p= 0.041) (Table 4, Table 4.1).

**Table 4:** odds of use of HMIS for healthcare decision making in Ketu North Municipal, 2020 (n=219)

Variables	Use of HMIS for healthcare decision making			
	Unadjusted ORs (95% CI)	P-value	Adjusted ORs (95% CI)	P-value
**Facility ownership**				
Private	Ref	-	-	-
Government	1.08 (0.89-3.67)	0.087	-	-
CHAG (Religious)	0.59 (0.23-1.41)	0.067	-	-
**Facility type**				
CHPS	Ref	-	-	-
Health centre	0.89 (0.28-1.78)	0.610	-	-
Clinic	1.56 (0.90-2.76)	0.070	-	-
Hospital	0.76 (0.41-2.11)	0.591	-	-
**Category of Health staff**				
Medical Doctor	Ref	-	-	-
Physician Assistant	1.32 (0.89-3.68)	0.211	-	-
Midwife	0.67 (0.21-1.65)	0.542	-	-
Nurse	2.76 (1.55-4.07)	0.091	-	-
Health Information Officer	0.56 (0.15-2.81)	0.071	-	-
Disease Control Officer	0.61 (0.12-1.99)	0.134	-	-
Mental Health Officer	2.41 (1.32-4.92)	1.013	-	-
Others	2.15 (1.02-3.78)	0.413	-	-
**Access to HMIS in facility or sub-district**				
Have no access	Ref	-	-	-
Have access	1.79 (1.02-3.40)	0.519	-	-
**Work experience of staff**				
6 months but <1year	Ref	-	-	-
1 year but <3 years	0.61 (0.23-1.15)	0.071	-	-
3 years but <5 years	1.24 (0.91-1.87)	0.089	-	-
5 years and above	0.46 (0.15-1.02)	0.656	-	-

**Table 4.1:** odds of use of HMIS for healthcare decision making in Ketu North Municipal, 2020 (n=219)

Variables	Use of HMIS for healthcare decision making	Variables	Use of HMIS for healthcare decision making	Variables
**Knowledge on reporting forms**				
Unable to mention a form	Ref	-	-	-
Able to mention at least a form	2.29 (1.92-3.79)	0.311	-	-
**Knowledge on importance of HMIS**				
Unable to mention any importance	Ref	-	-	-
Able to mention at least one importance	1.30 (0.88-2.97)	0.561	-	-
**Knowledge on reporting deadline**				
2^nd^ of the new month	Ref	-	-	-
3^rd^ of the new month	2.42 (1.94-4.15)	0.605	-	-
4^th^ of the new month	0.92 (0.09-1.51)	0.761	-	-
5^th^ of the new month	1.09 (0.87-3.47)	0.076	-	-
None of the above	2.22 (1.89-5.56)	0.901	-	-
**Availability of person responsible for data management**				
No	Ref	-	-	-
Yes	0.97 (0.50-1.09)	0.934	-	-
**Availability of data quality improvement team**				
No	Ref	-	-	-
Yes	0.71 (0.34-1.45)	0.341	-	-
**Training on computer use**				
No	Ref	-	-	-
Yes	2.57 (1.16-5.66)	0.020	2.19 (0.94-5.11)	0.070
**Feedback on data**				
No	Ref	-	-	-
Yes	2.40 (1.14-5.04)	0.021	3.91 (1.63-9.38)	0.002
**Knowledge on HMIS**				
High knowledge	Ref	-	-	-
Moderate knowledge	0.66 (0.26-1.64)	0.367	0.56 (0.21-1.50)	0.250
Low knowledge	1.78 (1.18-3.94)	0.046	2.56 (1.04-6.31)	0.041

P-value is significant if p < 0.05

## Discussion

The objective of this study was to assess the utilization of HMIS by sub-district health staff in Ketu North Municipality. The study revealed that the study participants had a high level of knowledge about Health Management Information Systems (HMIS), with higher utilization rates among those with 5 or more years of experience. Most participants were aware of reporting deadlines and understood HMIS's significance. Healthcare workers affiliated with CHAG facilities also showed higher HMIS utilization rates. Feedback on data usage is more effective for decision-making, and HMIS is used for planning work programs and daily activities.

The study found that participants generally had high knowledge about HMIS, with most correctly defining it. Also, they had good knowledge on different types of HMIS, with a higher percentage correctly identifying HAMs (77.6%) and DHIMS-2 (67.6%). However, most of the respondents lacked knowledge on reporting forms, as they could not mention any. One can therefore infer from the above findings that the study respondents had some good knowledge on HMIS. This corroborates findings of a similar study conducted by Abdul-Rahman in Yendi, northern Ghana which showed the study participants had good knowledge on HMIS and were able to capture medical records well [13]. Participants' considerable knowledge of HMIS can likely be attributed to their prior training by the Municipal Health Directorate on e-tracker, an integral part of DHIMS-2. E-tracker enables the capture of transactional data through electronic devices, and this training likely played a significant role in enhancing their understanding of HMIS [22,24]. Another study conducted by Rashida in the West Mamprusi district of Ghana observed a low level of knowledge on HMIS among health staff [19], contrary to our findings. The variation in outcomes might be explained by the difference in sample sizes; our study utilized a larger sample of 219 health staff, compared to Rashida's study where they only included 44 health staff.

The study found that respondents had a high level of knowledge about the utilization of HMIS, with higher utilization rates among those with 5 or more years of work experience. However, this was slightly lower than a 2017 study by Mboro [20] in Kenya, which found that 50.8% of participants had knowledge about HMIS utilization. The differences could be due to variations in study settings and work environments, potentially affecting exposure to HMIS. Most respondents were aware of the reporting deadline for the next level, but a significant proportion lacked awareness about HMIS's importance. This finding is also consistent with a study by Muhindo *et al*. [25] in Uganda, which also showed low knowledge of HMIS's importance. Our study found the differences in respondents´ knowledge on reporting forms, importance of HMIS, reporting deadline and utilization of HMIS to be statistically significant. The limited understanding of the significance of HMIS among the study participants could be attributed to several factors. One of the main reasons could be the absence of regular refresher training for most healthcare staff on HMIS. Also, newly recruited personnel with less than three years of experience in the field might not have received formal orientation on HMIS.

The study examined factors such as facility ownership, type, staff category, access to HMIS, and utilization of HMIS. Results showed that participants affiliated with CHAG facilities had higher HMIS utilization rates compared to those in government and privately-owned facilities. This was likely due to the substantial staff population in CHAG's two major facilities. Hospitals had the highest representation of staff, with varying degrees of HMIS utilization. Nurses also had the highest representation, with varying HMIS utilization rates. These findings suggest that factors such as facility ownership, staff type, and access to HMIS can influence HMIS utilization rates. Our study also found that respondents with access to Health Information System (HMIS) at their facilities had a higher utilization rate than those without access. This is consistent with a similar study in the West Mamprusi district of Ghana (19), which found that access to HMIS significantly influenced data usage. The study also found a significant association between health facility type, staff category, and HMIS utilization. However, there was no significant association between facility ownership and HMIS utilization. This could be due to the diversity among ownership categories, such as private, CHAG (Christian Health Association of Ghana), and government-owned health facilities, each with unique resources, infrastructure, and management approaches.

On the role of HMIS in healthcare decision-making, the results showed that healthcare workers with limited knowledge of HMIS were 2.6 times more likely to use it for decision-making than those with extensive knowledge. This difference may be due to their higher interest in learning about HMIS. The majority of respondents based their decisions on HMIS data feedback, but a smaller proportion did not use it. It also showed that healthcare workers who received feedback on data were 3.9 times more likely to use Health Information System (HMIS) for decision-making. This finding supports previous research in Rwanda, Ghana, Nigeria, and Kenya [6,20,25-27], which highlighted the importance of feedback as a crucial tool in HMIS utilization among health staff for informed decision-making. The study found that a majority of the respondents used Health Information System (HMIS) to plan their work programs and daily activities, consistent with some developing countries [6,27]. In a Kenyan study, district-level employees primarily relied on routine health data for program-related administration, particularly planning, monitoring, managing medical supplies, and handling medicines [20]. The study's limitations include its cross-sectional design, which may not establish temporal relationships and may be prone to recall bias, and its limited scope to Ketu North Municipality, which may not provide a general picture of HMIS utilization in the Volta Region. However, the large sample size ensures generalizability and corrections were made to enhance accuracy due to potential overestimation from participant self-reporting. The implications of these findings are that regular HMIS training for healthcare professionals is essential, along with mentorship programs for junior employees, reinforcement of feedback systems, and proper information about HMIS developments.

## Conclusion

The study revealed that healthcare staff have a high level of knowledge regarding HMIS and the associated reporting forms. Health staff also demonstrated a good understanding of the different types of HMIS. Those with more than 5 years of experience exhibited a high level of knowledge in HMIS utilization and reporting deadlines. Factors including health facility type, staff category, and access to HMIS within the facility do not significantly affect HMIS utilization. Participants with limited access to feedback tend to rely on the information primarily for decision-making purposes compared to other participants. To address these issues and improve HMIS implementation, continuous training for healthcare staff within facilities by the Ghana Health Service is recommended. This could lead to enhanced knowledge and utilization of HMIS, and result in more efficient data management, improved decision-making, and ultimately better healthcare service delivery.

### What is known about this topic

- Health Management Information Systems (HMIS) has substantially simplified data integration by combining data from several health programs into a common platform;
- The use of data within HMIS has grown in importance, particularly in policy formation and evidence-based decision-making processes;
- Despite advancement in HMIS, its full potential in Ghana remains untapped due to poor infrastructure, insufficient financing, incomplete/erroneous data, and knowledge gaps among users.

### What this study adds

- The study emphasizes a noteworthy finding that while the majority of health professionals have extensive knowledge of HMIS, application of the knowledge for decision-making is limited;
- It observed factors such as health facility type, staff category, and access to HMIS within the facility have no significant impact on HMIS utilization;
- The study's findings suggest that the availability of feedback plays a significant role in how participants approach decision-making.
